# Absence of GAPDH regulation in tumor-cells of different origin under hypoxic conditions *in – vitro*

**DOI:** 10.1186/1756-0500-2-8

**Published:** 2009-01-13

**Authors:** Harun M Said, Buelent Polat, Carsten Hagemann, Jelena Anacker, Michael Flentje, Dirk Vordermark

**Affiliations:** 1 Department of Radiation Oncology, Faculty of Medicine, University of Würzburg, Würzburg, Germany; 2 Tumor biology Laboratory, Department of Neurosurgery, Faculty of Medicine, University of Würzburg, Würzburg, Germany; 3 Department of Gynaecology and Obstetrics, Faculty of Medicine, University of Würzburg, Würzburg, Germany; 4 Department of Radiation Oncology, Faculty of Medicine, Martin-Luther-University-Halle-Wittenberg, Halle, Germany

## Abstract

**Background:**

Gene expression studies related to cancer diagnosis and treatment are important. In order to conduct such experiment accurately, absolutely reliable housekeeping genes are essential to normalize cancer related gene expression. The most important characteristics of such genes are their presence in all cells and their expression levels remain relatively constant under different experimental conditions. However, no single gene of this group of genes manifests always stable expression levels under all experimental conditions. Incorrect choice of housekeeping genes leads to interpretation errors of experimental results including evaluation and quantification of pathological gene expression. Here, we examined (a) the degree of GAPDH expression regulation in Hep-1-6 mouse hepatoma and Hep-3-B and HepG2 human hepatocellular carcinoma cell lines as well as in human lung adenocarcinoma epithelial cell line (A-549) in addition to both HT-29, and HCT-116 colon cancer cell lines, under hypoxic conditions *in vitro *in comparison to other housekeeping genes like β-actin, serving as experimental loading controls, (b) the potential use of GAPDH as a target for tumor therapeutic approaches was comparatively examined *in vitro *on both protein and mRNA level, by western blot and semi quantitative RT-PCR, respectively.

**Findings:**

No hypoxia-induced regulatory effect on GAPDH expression was observed in the cell lines studied *in vitro *that were; Hep-1-6 mouse hepatoma and Hep-3-B and HepG2 human hepatocellular carcinoma cell lines, Human lung adenocarcinoma epithelial cell line (A-549), both colon cancer cell lines HT-29, and HCT-116.

**Conclusion:**

As it is the case for human hepatocellular carcinoma, mouse hepatoma, human colon cancer, and human lung adenocarcinoma, GAPDH represents an optimal choice of a housekeeping gene and/(or) loading control to determine the expression of hypoxia induced genes in tumors of different origin. The results confirm our previous findings in human glioblastoma that this gene is not an attractive target for tumor therapeutic approaches because of the lack of GAPDH regulation under hypoxia.

## Background

Glyceraldehyde-3-phosphate dehydrogenase (GAPDH) is a glycolytic enzyme that possesses diverse functions that are independent of its role in glycolysis [1]. GAPDH is a multifunctional enzyme overexpressed in many tumors and induced by hypoxia in a number normal and malignant cells examined [1]. Experimental data previously published show that hypoxia induced transcriptional activation of GAPDH is cell-type specific [2]. GAPDH is considered to be a "housekeeping gene". Previous contributions showed that GAPDH expression is regulated by a variety of factors like calcium [3], insulin [4], and hypoxia [5], although the transcription factor hypoxia-inducible factor-1 (HIF-1) regulates the expression of genes which are involved in glucose supply, growth, metabolism, redox reactions and blood supply. The HIF family comprises the HIF-1α, HIF-1β, HIF-2α, and HIF-3α subunits [6]. Under normoxic conditions, the HIF-1α subunit is undetectable because it undergoes rapid ubiquitination and proteosomal degradation [7,8].

Hypoxia is characterized by inadequate oxygen delivery to the tissue with a resulting imbalance between oxygen demand and energy supply [9]. As a consequence, HIF-1-regulated hypoxia-induced genes are transcribed [[10-14], and [15]]. Many of the proteins encoded by these genes are involved in adaptive responses counteracting a detrimental impact of hypoxia, including erythropoiesis, angiogenesis, iron homeostasis, glucose and energy metabolism, as well as cell proliferation and survival decisions [10].

In human tumors, two types of hypoxia are present, transient and chronic hypoxia. Transient hypoxia is a tumor oxygenation status where a temporary reduction in oxygen availability is present. The inadequate vascular geometry relative to the volume of oxygen-consuming tumor cells creates diffusion-limited O_2 _delivery, which results in chronic hypoxia [16,17]. Cells in the hypoxic environment shift from aerobic citric acid cycle (TCA cycle) to anaerobic metabolism (glycolysis, also known as Warburg effect), as a result of chronic hypoxic conditions. The response to low O_2 _levels is given by up-regulating the synthesis of HIF [6]. Tumor microenvironment typically contains hypoxic regions, since tumor vasculature is dysfunctional and unable to meet the metabolic needs of rapidly proliferating cancer cells [18]. Tumor cells are resistant to therapeutic approaches, like ionizing radiation and chemotherapy. For ionizing radiation the dose required to produce the same amount of cell killing is up to three times higher for hypoxic cells than for well-oxygenated cells [19]. In glioblastoma, the modification of tumor oxygenation and thus radiosensitivity is an attractive approach to improve the prognosis of glioblastoma patients currently tested in clinical trials [20].

As previously shown [5,21,22], GAPDH expression increases as a response to the hypoxic development in endothelial cells. GAPDH regulation by hypoxia appears in a cell – type specific manner. From previous results, it was evident that GAPDH expression is induced to a much lesser extent in fibroblasts and smooth muscle cells than it is in endothelial cells [23].

In the present study we addressed the question whether GAPDH expression is up – or down-regulated by hypoxia in tumor cells of different origin – *in vitro*. Also, besides our previous published data [24,25] showing that GAPDH is not regulated in human glioblastoma under hypoxic conditions, our findings increases the potential tumor types where GAPDH represent a suitable loading control under hypoxic conditions and further, confirm the hypothesis that GAPDH expression regulation under hypoxia is a cell-specific posttranscriptional event.

## Results

### GAPDH mRNA expression is not regulated by hypoxia, *in vitro *in cancer cell lines of different origin

**Figure 1:**
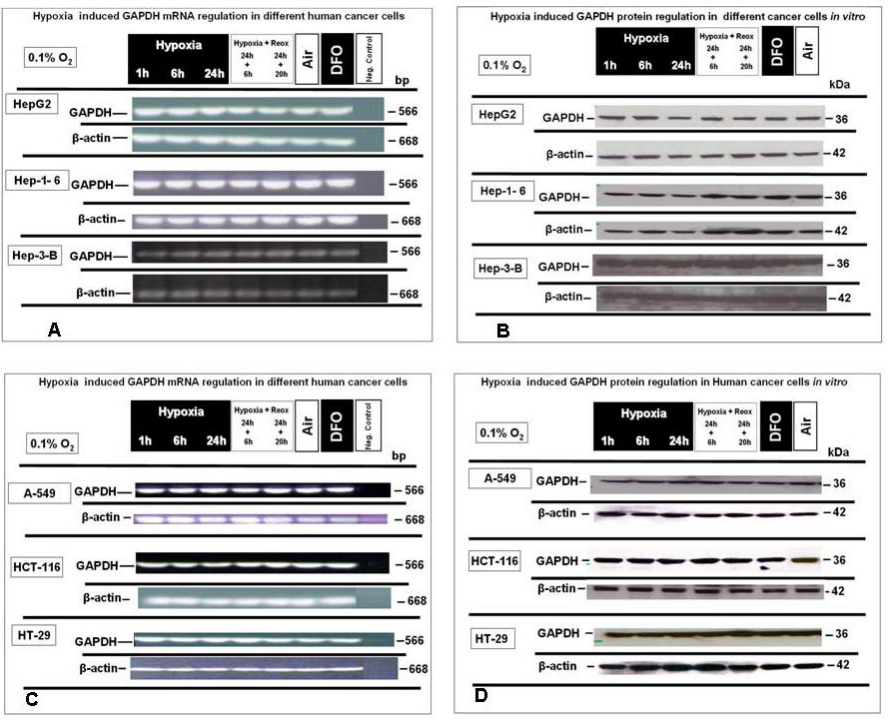
**GAPDH mRNA and protein expression under extreme hypoxic (0.1% O_2_), normoxia and reoxygenation conditions in HepG2, Hep 1-6, Hep-3-B, A-549, HT-29 and HCT-116 cells, *in vitro***. **(A) **Semiquantitative RT-PCR analysis of GAPDH mRNA expression in HepG2, Hep 1-6 and Hep-3-B **(B) **Western blot expression analysis of GAPDH protein under identical conditions in HepG2, Hep 1-6 and Hep-3-B. **(C) **Semiquantitative RT-PCR analysis of GAPDH mRNA expression in A-549, HT-29 and HCT-116, *in vitro ***(D) **Western blot analysis of GAPDH protein expression under identical conditions in A-549, HT-29 and HCT-116. Representative experiments out of three for each experimental set.

To examine that hypoxic conditions really do not regulate GAPDH expression, we performed *in vitro *cell culture assays with 0.1% O_2 _with and without re-oxygenation. No regulatory effect of these different oxygenation conditions on GAPDH expression was shown by semiquantitative RT-PCR in the Hep-1-6 mouse hepatoma and in Hep-3-B and HepG2 human hepato-cellular carcinoma cell lines (Fig. 1A) as well as in human lung adenocarcinoma epithelial cell line (A-549) and both HT-29 and HCT-116 colon cancer cell lines (Fig. 1C).

Densitometry analysis (see additional files 1, 2, 3, 4, 5) confirmed these results. Together, these data suggest that exposure of the tumor cells from different origin to an extreme hypoxic status (0.1% O_2_) is not associated with GAPDH mRNA up-regulation.

### Hypoxic conditions do not influence GAPDH protein expression *in vitro *in cancer cell lines from different origin

To exclude translational regulation of GAPDH protein expression by hypoxic conditions, western-blot analysis were performed using lysates from tumor cell lines treated and isolated under identical conditions, in parallel to those used for semi quantitative RT-PCR analysis. GAPDH and β-actin were detected and analysed in all samples from Hep-1-6.

Hep-3-B and HepG2 (Fig. 1B) as well as it was the case in protein lysates from (A-549), HT29 and HCT-116 (Fig. 1D). The data obtained from examination of the mRNA level GAPDH expression were confirmed, as protein expression of all two proteins was very homogeneously distributed and confirmed by densitometry.

### HIF-1α regulation in different tumor cells as a response to hypoxia

**Figure 2:**
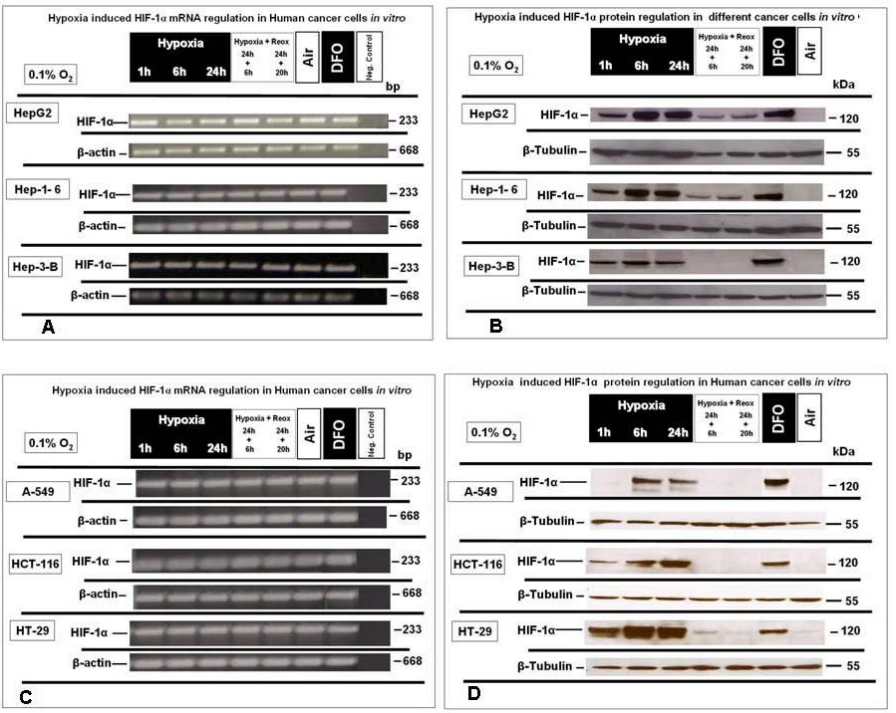
**HIF-1α-mRNA and -protein expression under extreme hypoxic (0.1% O_2_) and normoxia and reoxygenation conditions in HepG2, Hep 1-6, Hep3B, A-549, HT-29 and HCT-116 cells, *in vitro***. **(A) **Semi quantitative RT-PCR analysis of HIF-1α mRNA expression in HepG2, Hep 1-6 and Hep3B **(B) **Western blot analysis of HIF-1α protein expression under identical conditions in HepG2, Hep 1-6 and Hep3B. **(C) **Semi quantitative RT-PCR analysis of HIF-1α mRNA expression in A-549, HT-29 and HCT-116, *in vitro ***(D) **Western blot analysis of HIF-1α protein under identical conditions in A-549, HT-29 and HCT-116. Representative experiments out of three for each experimental set.

Semiquantitative RT-PCR analysis revealed that HIF-1α was evenly expressed at an oxygen concentration of 0.1% O_2 _for up to 24 h of hypoxia and continued a stable expression upon reoxygenation up to 20 h after 24 hours of hypoxia in Hep-1-6, Hep-3-B and HepG2 (Fig. 2A) as well as in (A-549), HT-29 and HCT-116 colon cancer cell lines (Fig. 2C), where there is no up-regulation of HIF-1α mRNA in the cell lines examined.

In contrast, and in parallel sets of experiments, HIF-1α nuclear protein expression was clearly up regulated under hypoxic conditions and down regulated under reoxygenation or normoxic conditions in Hep-1-6, Hep-3-B and HepG2 (Fig. 2B) as well as in A-549, HT-29, and HCT-116 (Fig. 2D). The results from nuclear protein expression analyses confirm oxygen-dependent regulation of HIF-1α at protein level, and reassure that the experimental settings for expression analysis of GAPDH were suitable to evaluate regulatory events by hypoxic conditions.

## Discussion

It has been postulated that GAPDH expression is regulated as a consequence of the hypoxic development of the cellular environment *in vitro *[[5,21-23], and [26]]. Several authors showed in their models that GAPDH mRNA expression was regulated during hypoxic events. Some also presented that the application of 18S-, 28S-RNA or β-actin instead as a loading control for experiments involving reduced oxygen concentration is more suitable for this purpose than GAPDH. Housekeeping genes are normally present in all cells and their expression levels should remain relatively constant under different experimental conditions. It is logic that no single housekeeping gene always possesses stable expression levels under all experimental conditions. Therefore, there is a necessity to characterize the suitability of various housekeeping genes to serve as internal RNA controls under particular experimental conditions where transcription effects are being tested.

To exclude a potential influence of oxygen concentrations on GAPDH expression as a confounding factor we have previously employed an additional control, 18S RNA, in experiments of hypoxia-inducible gene expression [6]. In these experiments, expression of GAPDH under different oxygen concentrations (severe hypoxia, normoxia and reoxygenation), was compared to the 18S RNA detected. They showed that GAPDH was not significantly regulated under hypoxic conditions in a panel of human tumor cell lines *in vitro*, and the expression of the gene examined was not altered after substitution of the GAPDH by the 18S RNA band with subsequent densitometric evaluation [21].

GAPDH induction by hypoxia in endothelial cells occurs via mechanisms other than those involved in other hypoxia-responsive systems [27]. A lack of regulation of GAPDH mRNA in response towards hypoxic events has also previously been demonstrated in the case of articular chondrocytes [28]. Table 1 summarizes literature data about GAPDH expression in response to the hypoxic development of the cellular environment by several tumor and non-tumor cells.

**Table 1:** Overview of GAPDH expression in different tumor and non-tumor cell lines as a consequence of the development of a hypoxic cellular microenvironment.

**Cell line or type**	**Origin**	**Genetic mutations**	**GAPDH overexpression**	**Ref**.	**Ref**.
		
		**(-/+)**	**under hypoxia**	**GAPDH**	**mutations**
**U373 – MG**	Malignant glioma cells (Human)	-Apoptosis resistant mutant P53	**(-)**	[24,25]	[40],
					
		-Peroxisome proliferator activated receptor – γ			
					
		-PTEN mutation			[41],

**GaMG**	Malignant glioma cells (Human)	**(-)**	**(-)**	[24,25]	[33], [41]

**U251**	malignant glioma cells (Human)	Mutated p53	**(-)**	[24,25]	[40]
					
		P14ARF/P16 deletion			

**Bovine articular chondrocytes**	Chondrocytes (Bovine)	Not determined	**(-)**	[37]	**(-)**

**LNCap**	Prostate adenocarcinoma cells	**(-)**	**(+)**	[34]	[27]
					
	(Human)				

**ATII**	Alveolar epithelial cells (Rat)	**(-)**	**(+)**	[26]	[26]

**SiHA**	Spontaneous cervical cancer cells (Human)	**(-)**	**(+)**	[35]	[36]
					
		Wild type p53			

**MBEC4**	Brain capillary endothelial cells	**(-)**	**(+)**	[37]	[37]
					
	(Mouse)				

**EC**	Endothelial cells (Human)	- Not determined	**(+)**	[5], [22], [23], [24,39], [39]	[39]
					
		- Mutated epithelial cells are present			

**HepG2**	Human Hepatocellular Carcinoma	Raf inactive (-)	**(-)**	[This Paper]	[42], [43]
					
		Wild type p53			
					
		Wild type Retinoblastoma			

**Hep-3-B**	Human Hepatocellular Carcinoma	Wild type p53	**(-)**	[This Paper]	[43,44]
					
		Absence of RB transcripts			
					
		Deletion p53 gene			

**Hep.1-6**	Mouse Hepatocellular Carcinoma	- Not determined	**(-)**	[This Paper]	[45]

**HCT-116**	Colon cancer cell line (Human)	**K-ras (+)**	**(-)**	**(-)**	[46]

**HT-29**	Colon cancer cell line (Human)	**(-)**	**(-)**	**(-)**	[46], [47]

**A-549**	Lung cancer cell line (Human)	**k-Ras (+)**	**(-)**	**(-)**	[47], [48]
					
		**c-RAS (+)**			

Our data did not reveal any correlation between hypoxia induced HIF-1α protein over expression and GAPDH regulation on mRNA and protein level *in vitro *in Hep-1-6 mouse hepatoma, Hep-3-B and HepG2 human hepatocellular carcinoma cell lines as well as in human lung adenocarcinoma epithelial cell line (A-549), in addition to both HT-29, and

HCT-116 colon cancer cell lines. Although we did not measure oxygenation levels directly in the human tumors, samples of which were analyzed regarding GAPDH expression, we considered low-grade astrocytoma and glioblastoma as tumor entities characterized by modest hypoxia and severe hypoxia, respectively. Based on these findings and from other studies, our present results suggest that there is also no hypoxia-dependent regulation of GAPDH in the tumor cells from different origin examined *in – vitro*.

## Conclusion

The appropriate choice of an internal standard is critical for quantitative protein and RNA analyses. However, no single housekeeping gene always manifests stable expression levels under all experimental conditions. Expression of GAPDH represents one of the alternatives of a housekeeping gene and can be used as a loading control in experiments with glioma cells as well as in human hepatocellular carcinoma, mouse hepatoma human colon cancer, and human lung adenocarcinoma. Regulation of GAPDH mRNA and protein expression as a response to the hypoxic development in the tumor cell environment is not an absolute phenomenon, but occurs as a cell-specific post-transcriptionally regulated event. Therapeutic strategies for treatment of hepatocellular carcinoma, human lung cancer or human colon involving GAPDH as target molecule do not represent a valid approach in conjunction with tumor hypoxia.

## Methods

### Cell and culture and hypoxia treatment

Early-passage human malignant lung cancer cell line A-549 as well as HT-29 and HT-116 from the American Type Culture Collection (ATCC, Rockville, MD) were grown on glass Petri dishes in Dulbecco's modified Eagle's medium, supplemented with 10% fetal bovine serum (FBS) and non-essential amino acids. Additionally, all culture media were supplemented with penicillin (100 IU/ml)/streptomycin (100 μg/ml) and 2 mM L-glutamine. Cells were treated with *in-vitro *hypoxia for 1, 6 or 24 hours at 0.1% O_2 _in a Ruskinn (Cincinnatti, OH, USA) Invivo_2 _hypoxic workstation as previously described [29-32]. For reoxygenation experiments, dishes were returned to the incubator after 24-hour hypoxia treatment.

### Preparation of cell lysates, nuclear cell extracts and immunoblotting

Cell lysates and nuclear extracts were prepared, stored and blotted using the similar techniques, antibodies, procedures and loading controls as conducted previously [25].

### Isolation of total RNA from tumor cell lines

Total RNA was isolated from cultured tumor cells as reported previously [6] and described in [25,31], including the digestion of contaminating DNA with the provided DNase, following the manufacturer's instructions.

### HIF-1α, β-actin and GAPDH mRNA expression levels in tumor cell lines from different origin by semiquantitative RT-PCR

To compare the expression of the individual genes examined, RT-PCR was performed using primers designed using published information on GAPDH, β-actin and HIF-1α mRNA sequences in GenBank (accession numbers NM_002046 for GAPDH, NM_001101 for β-actin and NM_001530.2 for HIF-1α, respectively). An aliquot of 1–5 μg of total mRNA from human glioblastoma and astrocytoma tissue or glioblastoma cell lines was transcribed at 42°C for 1 h in a 20 μl reaction mixture using 200 U RevertAid™ M-MuLV Reverse Transcriptase (RT), oligo(dT)18 primer and 40 U Ribonuclease inhibitor (all from Fermentas, Ontario, Canada).

For PCR-reactions primers were designed in flanking exons with Primer3 software (available online ): to produce an 566 bp amplification product of GAPDH, the forward primer (F1) was 5'-GCAGGGGGGAGCCAAAAGGG-3' (nucleotides 393 – 412) and the reverse primer (R1) 5'-TGCCAGCCCCAGCGTCAAAG-3' (nucleotides 939 – 958). To produce an 668 bp amplification product of β-actin, the forward primer (F1) was 5'-CGTGCGTGACATTAAGGAGA'-3 (nucleotides 697 – 716) and the reverse primer (R1) 5'-CACCTTCACCGTTCCAGTTT'-3 (nucleotides 1345 – 1364) and to produce an 233 bp amplification product of HIF-1α, the forward primer (F1) was 5'-TTACAGCAGCCAGACGATCA-3' (nucleotides 2516 – 2535) and the reverse primer (R1) 5'-CCCTGCAGTAGGTTTCTGCT-3' (nucleotides 2729 – 2748). The PCR was performed with 25 to 32 cycles with increments of 5 cycles using PCR systems and reagents acquired from Promega™ (Promega GmbH, Mannheim, Germany) and applied according to the manufacturer's instructions. The PCR products were separated on 2% agarose gels (Sigma-Aldrich, Steinheim, Germany) and visualized by ethidium bromide staining (0.07 μg/ml ethidium – bromide; Biorad, Munich, Germany).

### Densitometry

Signal strengths detection in western blots or in semi-quantitative RT-PCR was performed with 1D Kodak Image Analysis Software. The amount of RNA or proteins gave signals that were measured in Kodak light units (KLU) and divided by the corresponding signals of the loading control (β-tubulin and β-actin for western blots and semi-quantitative RT-PCR) as previously described [11,21]. 3–4 individual experiments were always performed. The Mann-Whitney U test for independent samples was used to analyse these data. P ≤ 0.05 was considered to be statistically significant. All tests were carried out using the statistical package SPSS, release 12.0.1 for Windows (SPSS Inc., Chicago, Ill., USA).

## Abbreviations

GAPDH: glyceraldehyde-3-phosphate dehydrogenase, Tumor hypoxia, β-actin, oxygen, glioblastoma multiforme, astrocytoma, HIF-1α, 18S RNA, house keeping gene.

## Competing interests

The authors declare that they have no competing interests.

## Authors' contributions

HMS was the primary author of the manuscript, performed the *in-vitro *hypoxia experiments, supplied the *in-vitro *mRNA, protein lysates and nuclear extracts, performed the Western blots, densitometric analysis of the results and participated in the study design. BP and CHco-authored the manuscript and participated in the study design. HMS, CH and BP coordinated the group and contributed to the development of the experimental strategy. JS designed the primers used for RT-PCR and participated in the study design and evaluation. CH, MF, and DValso participated in the study design. All authors read and approved the final manuscript.

## Supplementary Material

Additional file 1 **Additional files description (files 2–5, additional materials section).** Figures description of the densitometry evaluation results of GAPDH and HIF-1α protein and mRNA expression analysis, in the different tumor cells analyzed under different oxygenation conditions via western blots and RT – PCR, respectively. Click here for file

Additional file 2 **Densitometry evaluation of GAPDH mRNA expression.** Results of densitometry evaluation of GAPDH mRNA expression under different. oxygenation conditions in different tumor cells, detected via RT – PCR. Click here for file

Additional file 3 **Densitometry evaluation of GAPDH Protein expression.** Figure showing results of densitometry evaluation of GAPDH protein expression under different oxygenation conditions in different tumor cells, detected via western blot. Click here for file

Additional file 4 **Densitometry evaluation of HIF-1α mRNA expression.** Results of HIF-1α mRNA expression densitometry evaluation in tumor cells examined under different oxygenation conditions and detected via RT-PCR. Click here for file

Additional file 5 **Densitometry evaluation of HIF-1α Protein expression.** Results of densitometry evaluation of HIF-1α protein expression under different oxygenation conditions in the analyzed tumor cells detected via western blot. Click here for file
