# Supplementary material for: Absence of GAPDH regulation in tumor-cells of different origin under hypoxic conditions in – vitro
Source: BMC Res Notes. 2009 Jan 13;2:8. doi: 10.1186/1756-0500-2-8 (PMC2646737; doi:10.1186/1756-0500-2-8)
Supplement: Additional file 1 — Additional files description (files 2–5, additional materials section). Figures description of the densitometry evaluation results of GAPDH and HIF-1α protein and mRNA expression analysis, in the different tumor cells analyzed under different oxygenation conditions via western blots and RT – PCR, respectively. [file 1756-0500-2-8-S1.doc]

**Additional files description** **(files 2-5- additional materials section)**.

**- Additional file 2**

**Densitometric evaluation of GAPDH mRNA expression in tumor cells.**

Evaluation of GAPDH semi-quantitative RT-PCRs results from HepG2, Hep-1-6, Hep-3-B, A-549, HT-29 and HCT-116 mRNA lysates. Signal strength detection on agarose gels of semi-quantitative RT-PCRs was performed with 1D Kodak Image Analysis Software. The amount of RNA gave signals that were measured in Kodak light units (KLU) and divided by the corresponding signals of the loading control (β-actin for semi-quantitative RT-PCR) as previously described [11, 21].

**- Additional file 3**

**Densitometric evaluation of GAPDH Protein expression in tumor cells.**

Evaluation of GAPDH Western blot results from HepG2, Hep-1-6, Hep-3-B, A-549, HT-29 and HCT-116 protein lysates. Signal strength detection on Western blots was performed with 1D Kodak Image Analysis Software. The amount of protein gave signals that were measured in Kodak light units (KLU) and divided by the corresponding signals of the loading control (β-actin for Western blots) as previously described [11, 21].

**- Additional file 4**

**Densitometric evaluation of HIF-1α mRNA expression in tumor cells.**

Evaluation of HIF-1α semi-quantitative RT-PCRs results from HepG2, Hep-1-6, Hep-3-B, A-549, HT-29 and HCT-116 mRNA lysates. Signal strength detection on agarose gels of semi-quantitative RT-PCRs was performed with 1D Kodak Image Analysis Software. The amount of RNA gave signals that were measured in Kodak light units (KLU) and divided by the corresponding signals of the loading control (β-actin for semi-quantitative RT-PCR) as previously described [11, 21].

**- Additional file 5**

**Densitometric evaluation of HIF-1α protein expression in tumor cells.**

Evaluation of HIF-1α Western blot results from HepG2, Hep-1-6, Hep-3-B, A-549, HT-29 and HCT-116 protein lysates. Signal strength detection on Western blots was performed with 1D Kodak Image Analysis Software. The amount of protein gave signals that were measured in Kodak light units (KLU) and divided by the corresponding signals of the loading control (β-tubulin for Western blots) as previously described [11, 21].
