# Supplementary figures and images for: Absence of GAPDH regulation in tumor-cells of different origin under hypoxic conditions in – vitro
Source: BMC Res Notes. 2009 Jan 13;2:8. doi: 10.1186/1756-0500-2-8 (PMC2646737; doi:10.1186/1756-0500-2-8)

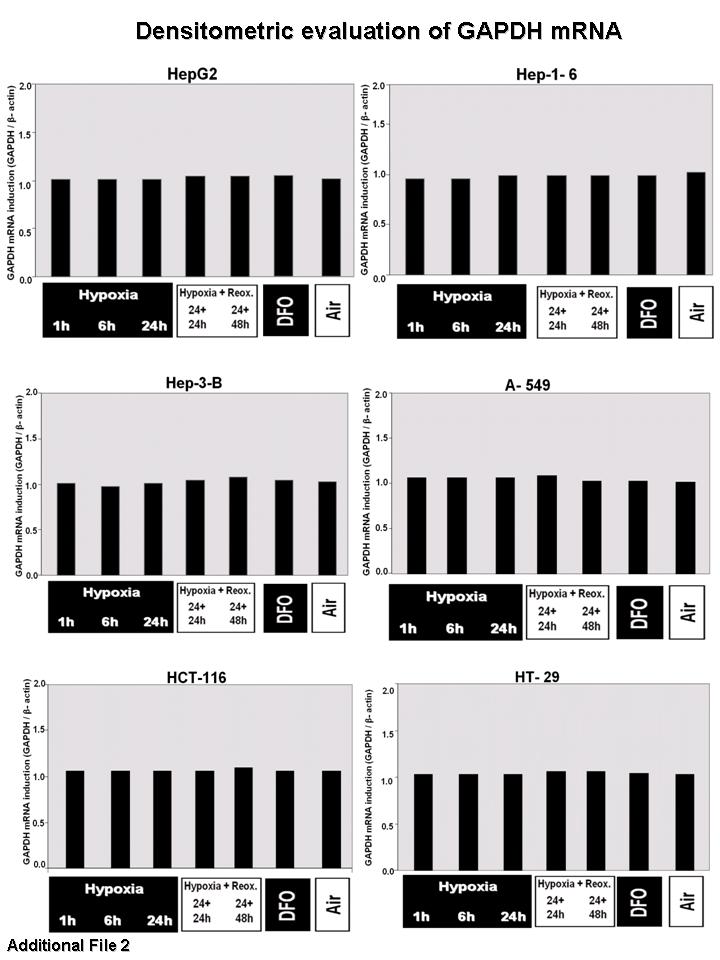

Supplement: Additional file 2 — Densitometry evaluation of GAPDH mRNA expression. Results of densitometry evaluation of GAPDH mRNA expression under different. oxygenation conditions in different tumor cells, detected via RT – PCR. [file 1756-0500-2-8-S2.jpeg]

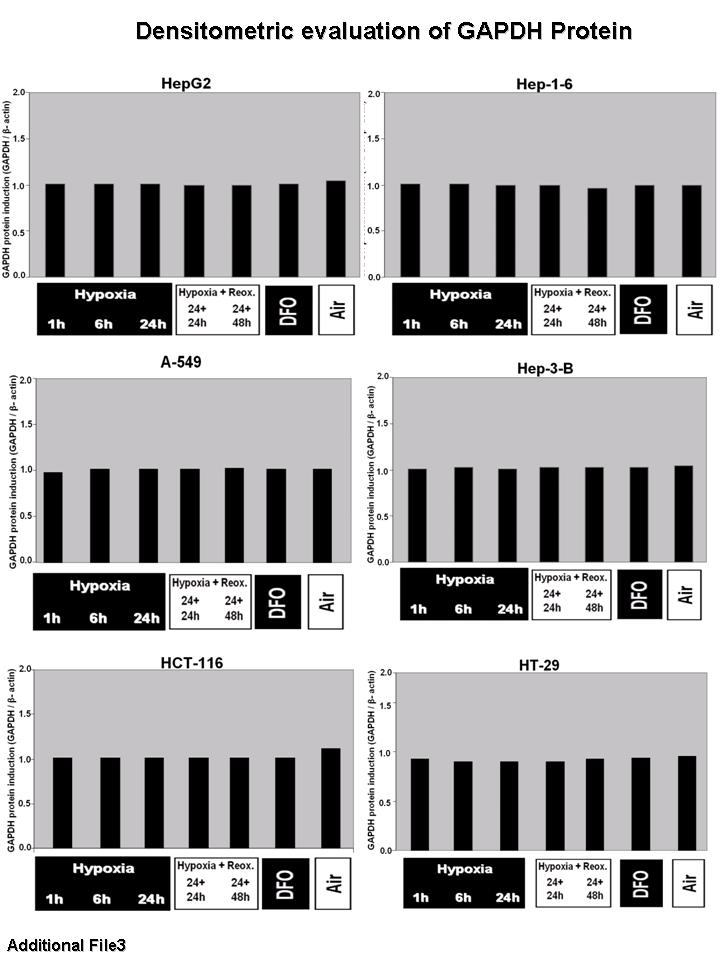

Supplement: Additional file 3 — Densitometry evaluation of GAPDH Protein expression. Figure showing results of densitometry evaluation of GAPDH protein expression under different oxygenation conditions in different tumor cells, detected via western blot. [file 1756-0500-2-8-S3.jpeg]

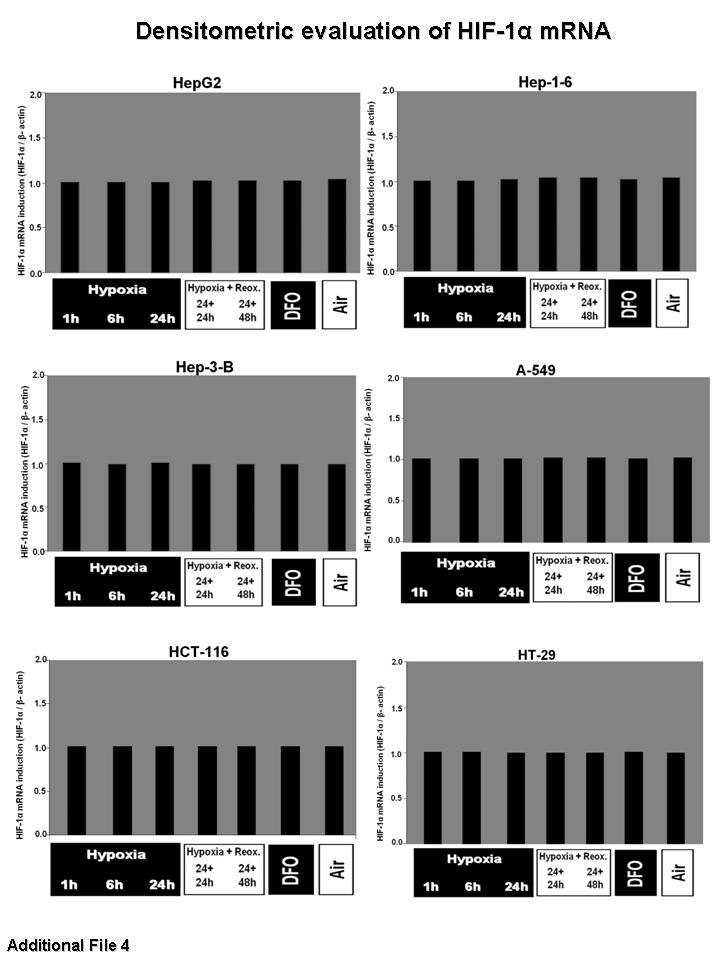

Supplement: Additional file 4 — Densitometry evaluation of HIF-1α mRNA expression. Results of HIF-1α mRNA expression densitometry evaluation in tumor cells examined under different oxygenation conditions and detected via RT-PCR. [file 1756-0500-2-8-S4.jpeg]

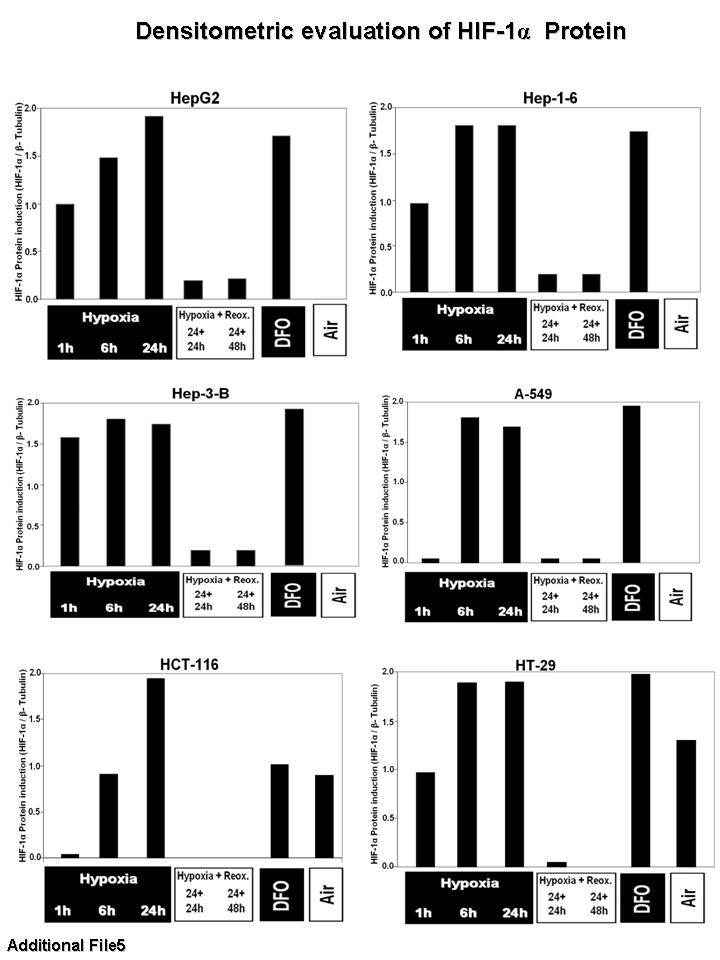

Supplement: Additional file 5 — Densitometry evaluation of HIF-1α Protein expression. Results of densitometry evaluation of HIF-1α protein expression under different oxygenation conditions in the analyzed tumor cells detected via western blot. [file 1756-0500-2-8-S5.jpeg]
